# Association of *ADRB2* polymorphism with triglyceride levels in Tongans

**DOI:** 10.1186/1476-511X-12-110

**Published:** 2013-07-23

**Authors:** Izumi Naka, Jun Ohashi, Ryosuke Kimura, Tsukasa Inaoka, Yasuhiro Matsumura

**Affiliations:** 1 Faculty of Medicine, University of Tsukuba,Tennodai 1-1-1, Tsukuba, Ibaraki 305-8575, Japan; 2 Transdisciplinary Research Organization for Subtropics and Island Studies, University of the Ryukyus, Nakagami, Okinawa 903-0215, Japan; 3 Department of Environmental Sociology, Faculty of Agriculture, Saga University, Saga 840-8502, Japan; 4 Faculty of Health and Nutrition, Bunkyo University, Chigasaki, Kanagawa 253-8550, Japan

**Keywords:** β2 adrenergic receptor gene, Body mass index, Polymorphism, Triglycerides

## Abstract

**Background:**

Our previous study demonstrated that the A-allele of the single nucleotide polymorphism (SNP) rs34623097 located in the upstream region of the β2 adrenergic receptor gene (*ADRB2*) is significantly associated with risk for obesity in Oceanic populations.

**Methods:**

To investigate whether the *ADRB2* polymorphisms explain part of the individual differences in lipid mobilization, energy expenditure and glycogen breakdown, the associations of 10 *ADRB2* SNPs with total cholesterol, high-density lipoprotein cholesterol, low-density lipoprotein cholesterol and triglyceride levels were examined in 128 adults in Tonga.

**Results:**

A multiple linear regression analysis adjusted for age, sex, and body mass index revealed that rs34623097 was significantly associated with triglyceride levels (*P*-value = 0.037). A copy of the rs34623097-A allele increased serum triglyceride levels by 70.1 mg/dL (0.791 mmol/L). None of the *ADRB2* SNPs showed a significant association with total-cholesterol, high-density lipoprotein cholesterol, or low-density lipoprotein cholesterol.

**Conclusions:**

In a Tongan population, a SNP located in the upstream region of *ADRB2* is associated with triglyceride levels independent of body mass index.

## Background

The β2 adrenergic receptor (ADRB2), a class of G protein-coupled receptor for catecholamines, plays an important role in regulating energy expenditure and lipolysis in adipose tissue. Therefore, polymorphisms in the *ADRB2* gene (OMIM*109690) may explain part of the individual differences in lipid profiles. The 27Glu allele at the Glu27Gln polymorphism of *ADRB2* has been significantly associated with increased triglyceride levels [1-6], although there is a conflicting report that demonstrated a significant increase in triglyceride levels in individuals with the Gln/Glngenotype as compared to those with the Glu/Glugenotype [7].

Most studies have examined the association of three non synonymous *ADRB2* SNPs, rs1042711 (5’LC-Arg19Cys in the 5’ upstream region), rs1042713 (Gly16Arg) and rs1042714 (Glu27Gln), with lipid profiles (i.e., total cholesterol, high-density lipoprotein [HDL] cholesterol, low-density lipoprotein [LDL] cholesterol and triglyceride levels). However, ourrecent study showed that rs34623097 located in the upstream region of *ADRB2* is more strongly associated with obesity than non synonymous SNPs in Oceanic populations. A functional analysis suggested that rs34623097-A, a risk allele for obesity, reduces the transcriptional activity of *ADRB2* as compared with rs34623097-G [8]. The aim of the present study is to further explore the association of *ADRB2* SNPs including rs34623097 with lipid profiles independent of body mass index (BMI) in adult Tongan subjects.

## Results

### Subject characteristics

A total of 128 unrelated subjects (40 men and 88 women) were recruited for the present study. The clinical and laboratory parameters of participants are summarized in Table 1. The median values of total cholesterol and triglycerides in Tongan male subjects exceeded the desirable ranges of less than 200 mg/dL for total cholesterol and less than 150 mg/dL for triglycerides. Age was significantly associated with total cholesterol and LDL cholesterol; sex and BMI were significantly associated with HDL cholesterol and triglycerides (Table 2).

**Table 1:** Clinical characteristics of subjects

**Characteristic**	**Overall**	**Male**	**Female**
	(n = 128)	(n = 40)	(n = 88)
Age	44 (33–53)	49 (36–55)	42 (32–51)
Height (cm)	167.4 (162.3-170.1)	172.5 (170.5-177.3)	164.2 (160.5-168.1)
Weight (kg)	96.9 (85.7-108.4)	100.1 (90.0-109.2)	96.3 (84.5-107.2)
BMI (kg/m2)	35.3 (31.2-39.3)	32.4 (29.9-36.5)	35.9 (31.5-40.1)
Total cholesterol (mg/dL)	199 (173–224)	203 (176–230)	197 (170–218)
HDL cholesterol (mg/dL)	45 (40–52)	42 (38–49)	47 (42–52)
LDL cholesterol (mg/dL)	128 (109–147)	125 (114–154)	129 (107–146)
Triglyceride (mg/dL)	107 (73–155)	152 (104–216)	99 (62–129)

Data are presented as median (interquartile range); *BMI* Body Mass Index, *HDL* High Density Lipoprotein, *LDL* Low Density Lipoprotein.

**Table 2:** Association of age, sex and BMI with lipid traits

**Independent variable**	**Dependent variable**											
	**Total cholesterol (mg/dL)**			**HDL cholesterol (mg/dL)**			**LDL cholesterol (mg/dL)**			**Triglyceride (mg/dL)**		
	**β**	**SE**	**P-value**	**β**	**SE**	**P-value**	**β**	**SE**	**P-value**	**β**	**SE**	**P-value**
Age (years)	1.1	0.33	**1.0 x 10**^**-3**^	0.0031	0.063	0.96	0.77	0.29	**8.6 x 10**^**-3**^	0.88	0.76	0.25
Sex (Male = 0, Female = 1)	−3.4	8.6	0.70	5.6	1.6	**8.9 x 10**^**-4**^	5.9	7.6	0.44	−94	20	**6.6 x 1**^**-6**^
BMI (kg/m2)	0.42	0.65	0.52	−0.47	0.12	**3.0 x 10**^**-4**^	0.60	0.57	0.30	3.4	1.5	**0.026**

A multiple regression analysis was performed to examine the association of age, sex, and BMI with each lipid trait; *BMI* Body Mass Index, *HDL* High Density Lipoprotein, *LDL* Low Density Lipoprotein.

### Association of *ADRB2* polymorphisms with lipid profiles

**Figure 1:**
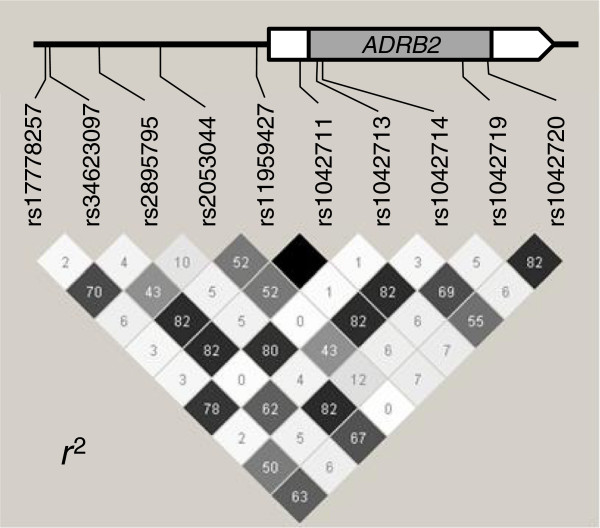
**LD structure of *ADRB2 *SNPs.** A pairwise *r*^2^ value is shown in each square. Darker shading indicates higher *r*^2^ values and black shading indicates an *r*^2^ of 1.

Two samples for rs11959427, three samples for rs1042713, and two samples for rs1042714 were not successfully genotyped by a molecular biology-based technique. To avoid a reduction in statistical power, these genotypes were imputed by using the MACH software [9]. Then we manually checked the imputed genotypes based on the LD structure (Figure 1), and concluded that the imputed genotypes seemed to be valid. The following analyses were therefore performed for the data including the imputed genotypes. No significant deviation from Hardy-Weinberg equilibrium was observed for the 10 *ADRB2* SNPs.

We adjusted the crude effect of each *ADRB2* SNP by taking into account age, sex and BMI, since these variables are significantly associated with lipid traits (Table 2). A multiple linear regression analysis adjusted for age, sex, and BMI indicated that rs34623097 was significantly associated with triglycerides in Tongan subjects (Table 3). A copy of the rs34623097-A allele, a risk allele for obesity in Oceanic populations [8], increased serum triglyceride levels by 70.1 mg/dL (0.791 mmol/L). No other significant associations were detected. It is noted that the derived allele, regardless of the allele frequency, was considered when determining the direction of association in Table 3.

**Table 3:** Association of each ADRB2 polymorphism with lipid traits

**SNP**	**Derived allele**	**Frequency**	**Total cholesterol (mg/dL)**			**HDL cholesterol (mg/dL)**			**LDL cholesterol (mg/dL)**			**Triglyceride**		
			**(mg/dL)**			**(mg/dL)**			**(mg/dL)**			**(mg/dL)**		
			**β**	**SE**	***P*-value**	**β**	**SE**	***P*-value**	**β**	**SE**	***P*-value**	**β**	**SE**	***P*-value**
rs17778257	T	0.39	5.17	5.52	0.351	0.110	1.06	0.918	4.34	4.83	0.371	8.20	12.8	0.523
rs34623097	A	0.04	13.7	14.6	0.350	−2.88	2.78	0.302	3.60	12.8	0.779	70.1	33.3	**0.037**
rs2895795	A	0.52	−8.09	5.42	0.138	−0.0671	1.04	0.949	−6.18	4.75	0.196	−19.1	12.5	0.130
rs2053044	A	0.09	10.0	9.82	0.312	−0.128	1.88	0.946	6.35	8.61	0.462	36.3	22.6	0.111
rs11959427a	T	0.95	−8.00	13.5	0.557	1.70	2.60	0.503	−3.50	11.9	0.768	−36.1	31.2	0.250
rs1042711 (Arg19Cys)	T (Cys)	0.95	−7.97	13.5	0.557	1.74	2.58	0.503	−3.51	11.9	0.768	−36.1	31.2	0.250
rs1042713 (Gly16Arg)^a^	A (Arg)	0.42	5.70	5.30	0.285	0.400	1.00	0.720	4.50	4.70	0.344	13.9	12.5	0.267
rs1042714 (Glu27Gln)^a^	C (Gln)	0.96	−8.50	14.6	0.563	2.10	2.80	0.443	−5.20	12.8	0.683	−37.9	33.7	0.263
rs1042719	C	0.43	4.42	5.38	0.413	−0.102	1.03	0.921	2.30	4.72	0.627	23.7	12.3	0.057
rs1042720	A	0.61	−2.47	5.48	0.652	0.616	1.04	0.556	0.05	4.80	0.992	−23.8	12.5	0.059

A multiple regression analysis adjusted for age, sex, and BMI was performed to examine the association of each polymorphism with each lipid trait; *BMI* Body Mass Index, *HDL* High Density Lipoprotein, *LDL* Low Density Lipoprotein.

^a^ The genotypes of subjects whose genotypes had not been determined by a molecular biology-based technique were imputed by using the MACH software.

In this study, besides nine tag SNPs (rs17778257, rs34623097, rs2895795, rs2053044, rs11959427, rs1042711, rs1042713, rs1042714, and rs1042720) of *ADRB2*, the rs1042719 SNP was further evaluated becausers 1042720, being in LD with rs1042719 in the Oceanic populations [8], showed a small *P*-value for triglyceride (*P*-value = 0.059; Table 3); however, the rs1042719 SNP was also not significantly associated with the level of triglycerides (*P*-value = 0.057).

**Figure 2:**
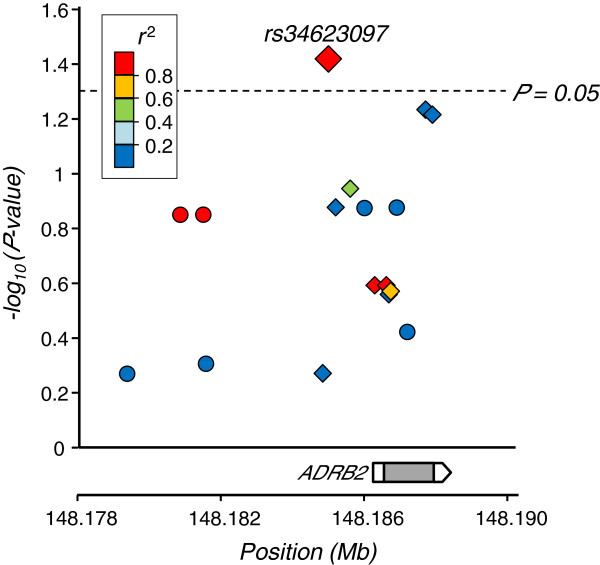
**Regional association plot.** The genotypes of SNPs around the *ADRB2* gene in Tongan subjects were imputed by the MACH software by using the genotype data of HapMap JPT and CHB samples as the reference panel. The association *P*-values for 10 genotyped and 7 imputed SNPs are plotted as diamonds and circles, respectively. The *r*^2^ between the SNP and rs34623097 is colored as a scale from low (blue) to high (red). In the genomic region, there is no protein-coding gene other than *ADRB2*.

To assess the possibility that the association of rs34623097 with triglycerides is caused by nearby SNPs being in LD with rs34623097, an additional multiple regression analysis was further performed for 7 SNPs imputed by the MACH software [9] by using the genotype data of HapMap East Asian populations (JPT + CHB) as the reference panel. No SNPs other than rs34623097 showed a significant association with triglycerides (Figure 2).

## Discussion

The present results indicate that rs34623097-A is associated with increased serum triglyceride levels. Our previous luciferase reporter assay demonstrated that rs34623097-A reduces the transcriptional activity of *ADRB2* as compared with rs34623097-G, and an electrophoretic mobility shift assay suggested that rs34623097 modulates the binding affinity with nuclear factors [8]. Taken together, these results indicate that the reduced expression of ADRB2 caused by rs34623097-A on adipocytes might contribute, in part, to increased serum triglyceride levels. One possible mechanism is that the activation of β adrenergic receptors such as ADRB2 expressed in adipocytes leads to the breakdown of triglycerides stored in adipocytes and the release of free fatty acids and glycerol [10-12]. Thus, the decreased lipolytic function in adipocytes is due to the lower expression of ADRB2 that would result in the accumulation of triglycerides within adipocytes. If triglycerides accumulate in adipocytes, the cellular uptake of the major component of triglycerides (i.e., free fatty acids) by adipocytes would be reduced. This mechanism would reduce the lipolysis of circulating lipoprotein-triglycerides. Accordingly, the level of serum triglycerides would be increased in individuals with rs34623097-A.

To the best of our knowledge, this study is the first to report that rs34623097 is a major *ADRB2* polymorphism that influences the serum triglyceride level, although the possibility that the lack of association of the other *ADRB2* polymorphisms with lipid profiles comes from the small sample size (n = 128) should not be excluded. Previous studies have revealed that the 27Glu allele (rs1042714-G) of *ADRB2* significantly increases the serum triglyceride levels [1-6]. In the present study, 27Glu also showed the same tendency (i.e., the β coefficient of 27Gln, an alternative allele at Glu27Gln, was a negative value in Table 3). This tendency is because rs34623097-A, which is significantly associated with increased triglycerides, is in positive LD with 27Glu in the Tongan subjects (*D*’ = 1 and *r*^2^ = 0.62). The rs34623097-A allele is observed mainly in Asians and Pacific Islanders and is in LD with 27Glu [8]. However, rs34623097-A had never previously been examined in the association studies on lipid traits including triglycerides. The significant association of 27Glu found in previous studies may merely reflect the LD with rs34623097-A. Further studies are required to clarify this issue in various ethnic groups.

## Subjects and methods

### Subjects

A total of 128 healthy adult subjects (18 years old or older) were recruited from Nuku’alofa, Tonga. Patients with diabetes and subjects who had any treatment known to interfere with metabolic syndrome-related parameters were excluded. A blood sample was collected from each subject after obtaining a written consent to participate in the study. This study was approved by the National Health Ethics & Research Committee of Tonga and the Research Ethics Committee of the Faculty of Medicine, University of Tsukuba.

### Anthropometric measurements

Anthropomorphic phenotypes were directly measured in field settings. Measurements were taken of subjects dressed in light clothing. Body height was measured to the nearest 1 mm by using a field anthropometer (GPM, Zurich, Switzerland), and weight was recorded to the nearest 0.1 kg by using a portable digital scale (Tanita model BC-518, Tokyo, Japan). BMI was calculated by dividing the weight in kg by the height in meters squared.

## Methods

Blood samples were obtained on the morning following a 12-hour fast. Serum lipids including total cholesterol, HDL cholesterol, LDL cholesterol, and triglycerides were measured at SRL Inc. (Tokyo, Japan) by using standard laboratory protocols.

### Genotyping

Genomic DNA was extracted from peripheral blood by using a QIAamp Blood Kit (Qiagen, Hilden, Germany). Nine tag SNPs (rs17778257, rs34623097, rs2895795, rs2053044, rs11959427, rs1042711, rs1042713, rs1042714, and rs1042720) of *ADRB2*, which were in LD (*r*^2^ > 0.8) with the other *ADRB2* SNPs in the Oceanic populations, were genotyped in our previous study [8]. The rs1042719 SNP was genotyped by using a TaqMan SNP genotyping assay in the present study because rs1042720, being in LD with rs1042719, showed not a significant but a small *P*-value in the association analyses of triglycerides in a Tongan population.

### Statistical analysis

Associations of age, sex, and BMI with total-cholesterol, HDL-cholesterol, LDL-cholesterol or triglyceride levels were assessed by a multiple regression analysis. The genotypes of rs11959427, rs1042713, and rs1042714 were not determined by a molecular biology-based technique for some subjects; instead, their genotypes were imputed by the MACH software [9]. Deviation of genotype frequencies from Hardy-Weinberg equilibrium was examined by chi-square test. Pairwise linkage disequilibrium (LD) parameters, *D*’ and *r*^2^, were estimated by using Haploview software [13]. The association of each polymorphism with total cholesterol, HDL cholesterol, LDL cholesterol or triglyceride levels was assessed by a multiple regression analysis adjusted for age, sex, and BMI. In the regression analysis, the number of copies of a derived allele at each SNP was used as an independent variable (i.e., homozygotes of an ancestral allele, homozygotes of a derived allele, and heterozygotes were coded as 0, 2, and 1, respectively). The genotypes of SNPs with minor allele frequency (MAF) of ≥ 0.05 spanning a 200 kb genomic region containing the entire *ADRB2* gene were retrieved from the HapMap JPT (Japanese in Tokyo, Japan) and CHB (Han Chinese in Beijing, China) populations [14,15]. Furthermore, genomic DNA samples from 43 JPT and 45 CHB subjects were obtained from the Coriell Cell Repository and subjected to rs34623097 genotyping [8]. By using the genotype data of JPT and CHB subjects as a reference, the genotypes of Tongan subjects were imputed by the MACH software [9]. The imputed genotypes of SNPs showing an Rsq value (a measure which estimates the squared correlation between imputed and true genotypes) of more than 0.5 were used for the association test. Accordingly, seven imputed SNPs were further subjected to single-point association analysis. *P*-values less than 0.05 were regarded as statistically significant.

## Conclusions

In a Tongan population, the rs34623097-A allele at a SNP located in the upstream region of *ADRB2* is significantly associated with increased serum triglyceride levels independent of BMI.

## Competing interests

The authors declare that they have no competing interests.

## Authors’ contributions

IN and JO performed statistical analyses and wrote the manuscript. IN and RK extracted DNA. IN performed genotyping. JO, RK, TI, and YM measured anthropomorphic phenotypes and collected blood samples. TI and YM contributed acquisition of lipid profile data. IN, JO, RK, TI, and YM participated in the design and coordination of the study. All authors read and approved the final manuscript.
